# Prognostic significance of age related genes in patients with lower grade glioma

**DOI:** 10.7150/jca.41123

**Published:** 2020-04-06

**Authors:** Haiwei Wang, Xinrui Wang, Liangpu Xu, Ji Zhang, Hua Cao

**Affiliations:** 1 Fujian Key Laboratory for Prenatal Diagnosis and Birth Defect, Fujian Maternity and Child Health Hospital, Affiliated Hospital of Fujian Medical University, Fuzhou, Fujian, China.; 2 Key Laboratory of Technical Evaluation of Fertility Regulation for Non-human Primate, National Health and Family Planning Commission, Fuzhou, Fujian, China.; 3 State Key Laboratory for Medical Genomics, Shanghai Institute of Hematology, Rui-Jin Hospital Affiliated to School of Medicine, Shanghai Jiao Tong University, Shanghai, China.

**Keywords:** Age related genes, Lower grade glioma, EMP3, SERPINE1, TCGA

## Abstract

**Objective**: To analyze the prognostic effects of age in different tumor types and determine the prognostic significance of age related genes in patients with lower grade glioma (LGG).

**Methods**: The relationships between age and tumor overall survival were determined by Kaplan-Meier survival analysis using The Cancer Genome Atlas (TCGA) dataset. The age related genes were identified using TCGA RNA-seq data. Univariate and multivariate cox regression were used to determine the prognostic significance of age related genes. The results derived from TCGA dataset were further validated using Gene Expression Omnibus (GEO) and Chinese Glioma Genome Atlas (CGGA) datasets.

**Results**: Age at initial pathologic diagnosis was most associated with the overall survival of LGG patients than other types of tumor patients. Age related genes EMP3, IGFBP2, TIMP1 and SERPINE1 were highly expressed in old LGG patients. The hypo-methylations of EMP3 and SERPINE1 were contributing to the high expressions of EMP3 and SERPINE1 in old LGG patients. Also, EMP3, IGFBP2, TIMP1 and SERPINE1 were highly expressed in LGG tumor tissues, compared with normal brain tissues. Moreover, high expressions of IGFBP2, EMP3, TIMP1 and SERPINE1 were associated with the worse prognosis of LGG patients. Furthermore, we demonstrated that EMP3 and SERPINE1 were connected with each other and the combination of EMP3 and SERPINE1 had better prognostic effects in glioma patients.

**Conclusions**: Age related genes IGFBP2, EMP3, TIMP1 and SERPINE1 have significant prognostic effects in LGG patients.

## Introduction

Cancer is an age related disease. With the increasing of age, somatic mutations are accumulating, contributing to the high incidence of cancer in older adults 1-4. Moreover, old tumor patients demonstrate different histological characteristics and prognostic outcomes as compared with young tumor patients 5-12. Those observations highlight the prognostic importance of age of tumor patients at initial pathologic diagnosis. However, the prognostic effects of age across different tumor types and the age related genes in determining the clinical outcomes are unclear.

For the decade, The Cancer Genome Atlas (TCGA) program collects the clinical characteristics and molecular signatures of more than 11,000 human tumor patients across 33 different tumor types 13, 14. Molecular profiles, like gene expression portraits, DNA mutation signatures and DNA methylation characteristics are quite associated with the clinical overall survival of tumor patients 15-19. Similar dataset is established in the Chinese Glioma Genome Atlas (CGGA) to provide massive amounts of data for basic and clinical research of glioma 20-22. With the available TCGA, CGGA and Gene Expression Omnibus (GEO) datasets, here, we systematically define the molecular characteristics of age related genes and reveal their prognostic effects in determining the clinical overall survival of tumor patients, particularly LGG patients.

Glioma is the most common type of brain tumor 23. According to the World Health Organization classification system, LGG is grade II- III glioma, contrast to the high grade glioblastoma (GBM) (grade IV glioma) 24. GBM and LGG demonstrate different clinical and molecular characteristics 25, 26. Unlike other tumor types, LGG is more likely developed in younger patients 27. Indeed, nearly 90% LGG patients are developed under 60 years old, according to the TCGA clinical data. Furthermore, our results show that the initial pathologic diagnostic age is most significantly associated with the tumor incidence and clinical overall survival of LGG patients compared with other types of tumor patients.

Previous results suggested that EMP3 28-32 and IGFBP2 33-35 were critical biomarkers in glioma prognosis. However, the inner mechanisms of how EMP3 and IGFBP2 involving the glioma overall survival are unclear. In here, we show that both EMP3 and IGFBP2 are age related genes, and highly expressed in old LGG patients. Additionally, we identify two prognostic genes TIMP1 and SERPINE1. Similar to EMP3 and IGFBP2, TIMP1 and SERPINE1 are highly expressed in old LGG patients. And IGFBP2, EMP3, TIMP1 and SERPINE1 are all highly expressed in LGG tumor tissues compared with normal brain tissues. Moreover, higher expressions of IGFBP2, EMP3, TIMP1 and SERPINE1 are associated with worse prognosis of LGG patients. At last, we demonstrate the prognostic effects of combination of EMP3 and SERPINE1 genes in glioma patients.

Taken together, our results suggest that age related genes IGFBP2, EMP3, TIMP1 and SERPINE1 have significant prognostic effects in LGG patients and represent suitable biomarkers for prognostic or therapeutic strategies for LGG patients.

## Materials and Methods

### Data collection

The TCGA datasets were downloaded from the TCGA hub (tcga.xenahubs.net), where all TCGA clinical data and molecular data were available. In the TCGA clinicalMatrix dataset, solid primary tumor patients with age at initial pathologic diagnosis and overall survival time were selected for further studies. Tumor types with favorable clinical outcomes, like kidney chromophobe (KICH), thyroid cancer (THCA) were excluded, since most of tumor patients were survived during the following time.

The gene expression series matrix of normal and cancerous brain tissues was downloaded from the Gene Expression Omnibus (GEO) website (www.ncbi.nlm.nih.gov/geo), and included the GEO datasets GSE4920 36, GSE16011 37 and GSE50161 38. The gene expression and survival data of patients with glioma were downloaded from the GEO dataset GSE43378 39.

The Chinese Glioma Genome Atlas (CGGA) datasets were downloaded from http://www.cgga.org.cn/index.jsp website, where all CGGA clinical data and gene expression data were available.

### TCGA data processing

Gene expression profile between old and young tumor patients of each tumor type was analyzed using TCGA RNA-seq dataset. The different gene expression between old and young tumor patients was determined by Student's t test. The DNA methylation profile of age related genes in glioma patients was analyzed through TCGA Human Methylation450 microarray dataset.

### GEO data processing

The GEO expression datasets were processed using R software. The matrix file of each dataset was annotated with corresponding platform. When multiple probes corresponded to the same gene symbol, the expression values were averaged using “plyr” package R software. The different gene expression between normal and glioma samples was determined using Student's t test.

### Heatmap presentation

Heatmaps were created by R software “pheatmap” package. The “pheatmap” package and the basic usage were downloaded from bioconductor. The clustering scale was determined by "average" method.

### Survival analysis

The overall survival curves were generated from prims5.0. Kaplan-Meier estimator was applied to determine the significance of different factors in determining tumor overall survival. P values were determined using Log-rank test.

### The construction of transcriptional network

The transcriptional network of age related genes was created by Cytoscape GeneMANIA App. Cytoscape is an open source software platform for constructing complex networks and could be download from Cytoscape website.

### Univariate and multivariate cox regression

Univariate and multivariate cox regression were analyzed by R software “survival” package. The “survival” package and the basic usage were downloaded from bioconductor. Log-rank test was used to calculate the P values.

### Venn diagram

The Venn diagram was generated using VENNY 2.1. VENNY 2.1 is an online tool for comparing lists (https://bioinfogp.cnb.csic.es/tools/venny/).

### Spearman correlation

Spearman correlation was used to study the correlation between EMP3 and SERPINE1 gene expression in glioma patients through the “lm” method in R software.

### Statistical analysis

The box plots were generated from prims5.0. Statistical analysis was performed using the Student's t test. P value less than 0.05 was chosen to be significantly different.

## Results

### Older age is associated with the low overall survival of tumor patients

Age at initial pathologic diagnosis and tumor overall survival were extracted from the clinicalMatrixs which were launched by the TCGA network. Totally, 8,810 tumor patients from 20 tumor types were collected for further studies. The abbreviations of tumor types and the number of tumor patients in each tumor type were illustrated in Table 1. The abbreviation of each tumor type was further used. The detailed clinical parameters of 8,810 tumor patients, including gender, weight, height, pathologic stage were provide in supplementary table.

**Figure 1:**
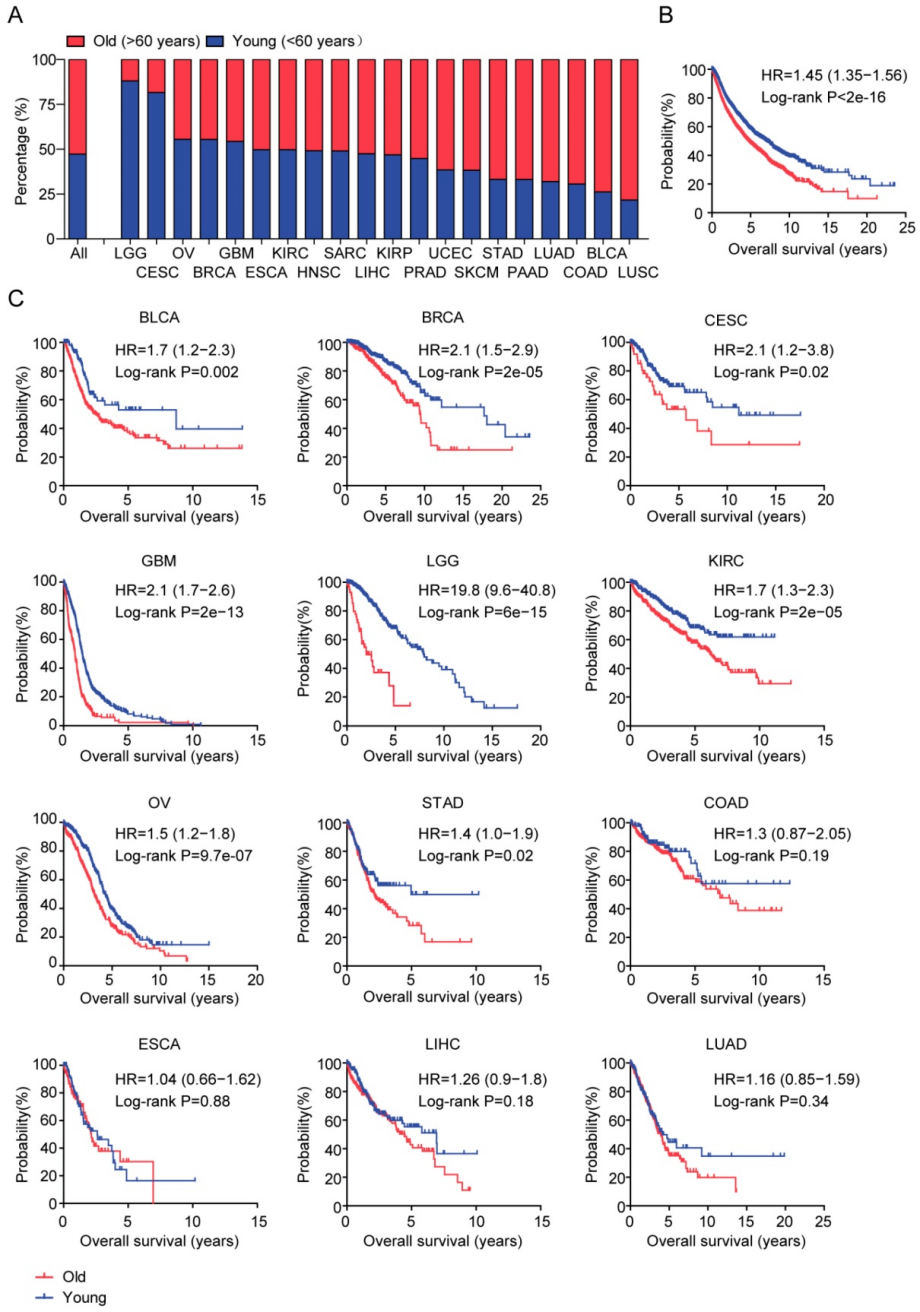
Age is most associated with the overall survival of LGG patients than other types of tumor patients. (A) Box plot showed the percentage of old (red) and young patients (blue) across 21 tumor types. (B) Overall survival was analyzed in old (red) and young patients (blue) across 21 tumor types. P value was generated from Log-rank test. (C) Overall survival was analyzed in old (red) and young patients (blue) in each 12 individual tumor type.

Collectively, the mean age of those tumor patients was 60.58 years old. Based on the age at initial pathologic diagnosis, tumor patients were divided into old (older than 60 years) and young group (equal or younger than 60 years). There were 4,169 tumor patients in old group and 4,641 tumor patients in young group (Table 1). Although, generally, cancer was an old related disease, we showed that some tumor types were preferentially developed in young adults. Particularly, more than three quarter of LGG and CESC patients were younger than 60 years (Figure 1A), while, more than three quarter of BLCA and LUSC patients were older than 60 years (Figure 1A).

Next, we tested the associations between age and tumor overall survival. The Kaplan-Meier survival analysis showed that tumor patients in old group had lower overall survival compared with the tumor patients in young group (P<2e-16) (Figure 1B). The median survival time of young tumor patients was 7.05 years, much longer than the 4.76 years of old tumor patients.

### Age is most associated with the overall survival of LGG patients than other types of tumor patients

Next, we studied the association between age and tumor overall survival in each tumor type. The Kaplan-Meier survival analysis demonstrated that age at initial pathologic diagnosis was a critical prognostic factor in 8 out of 21 tumor types, including BLCA, BRCA, CESC, GBM, LGG, KIRC, OV and STAD (Figure 1C). Particularly, age was most correlated with the overall survival of LGG patients (P=6e-15 and HR=19.8) (Figure 1C). The median survival time of old LGG patients was 1.99 years, contrast to the 7.88 years in young LGG patients. Although not as significant as LGG, the overall survival of old BCAL, BRCA, CESC, GBM, KIRC, OV and STAD patients were also significantly lower than young BCAL, BRCA, CESC, GBM, KIRC, OV and STAD patients (Figure 1C). However, age at initial pathologic diagnosis was not a prognostic factor in other tumor types, like COAD, ESCA, LIHC and LUAD (Figure 1C).

Our results suggested that, in most of tumor types and across tumor types, age at initial pathologic diagnosis was a critical prognostic factor. Particularly, age was most associated with the overall survival of LGG patients than other types of tumor patients.

### Identification of age related genes in TCGA dataset

**Figure 2:**
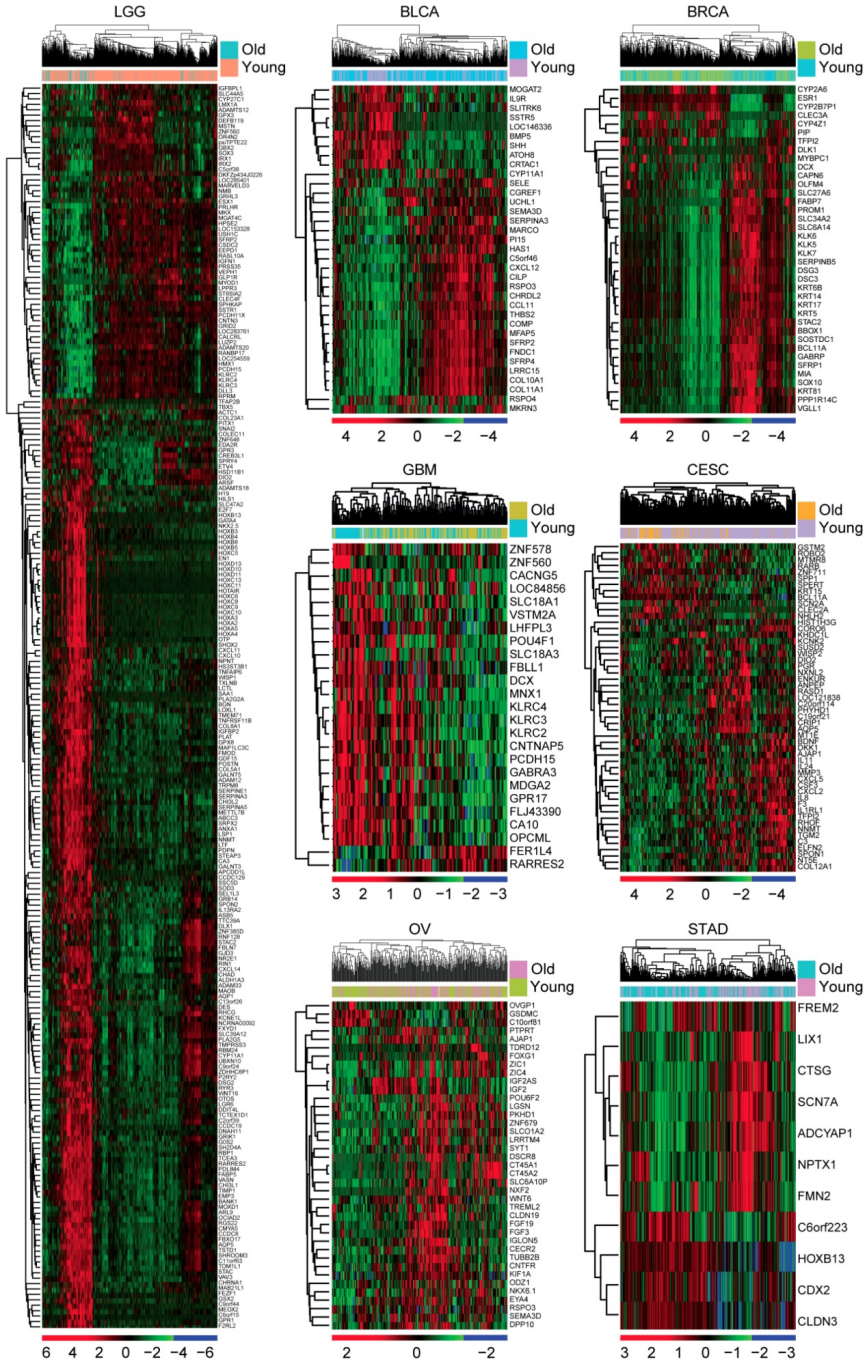
Identification of age related genes in TCGA dataset. Unsupervised clustering heatmaps demonstrated the differentially expressed genes (fold change >2) in old and young LGG, BLCA, BRCA, GBM, CESC, OV and STAD patients.

Next, we tried to determine the inner mechanisms of how age influenced the overall survival of tumor patients. Gene expression profiles of old patients in each age related tumor type were analyzed. Compared with the young tumor patients, genes up or down regulated in old tumor patients were identified respectively (fold change >2). We found that 230 genes were differentially expressed in old LGG patients (Figure 2). However, only 25 genes were differentially expressed in old GBM patients and 11 genes were differentially expressed in old STAD patients (Figure 2). Compared with young BLCA, BRCA, CESC and OV patients, genes differentially expressed in old BLCA, BRCA, CESC and OV patients were also identified (Figure 2). Consistent with the previous result that age was most associated with the overall survival of LGG patients than other types of tumor patients, we found that there were most age related genes in old LGG patients. So, in our further studies, we focused on LGG tumor type.

### Validation of age related genes in glioma patients derived from GEO dataset

From the TCGA dataset, we found that old LGG patients were associated with low overall survival, and 230 genes were differentially expressed in old LGG patients. To further confirm those findings, we analyzed the functions of age related genes from GEO datasets.

**Figure 3:**
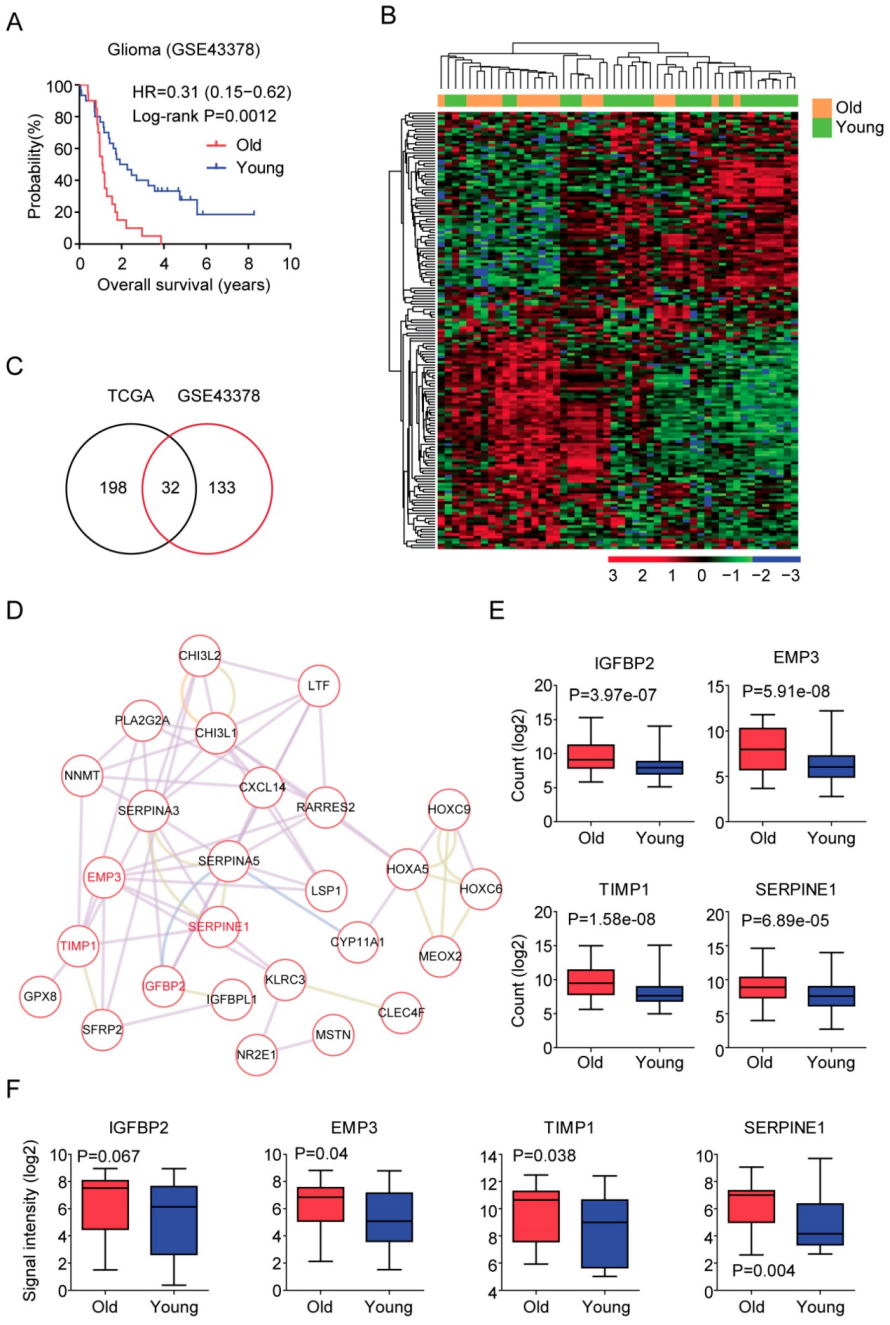
Validation of age related genes in glioma patients derived from GEO dataset. (A) Overall survival was analyzed in old (red) and young (blue) glioma patients in GSE43378 dataset. P value was generated from Log-rank test. (B) Unsupervised clustering heatmap demonstrated the differentially expressed genes (fold change >2) in old and young glioma patients in GSE43378 dataset. (C) Venn diagram depicted the common differentially expressed genes from TCGA and GSE43378 datasets. (D) Regulatory network was created by cytoscape using the 32 age related genes. (E) Box plots showed the IGFBP2, EMP3, TIMP1 and SERPINE1 expressions (log2 normalization count) in TCGA LGG patients. (F) Box plots showed the IGFBP2, EMP3, TIMP1 and SERPINE1 expressions (log2 signaling intensity) in GSE43378 glioma patients.

GSE43378 included gene expression, age at initial pathologic diagnosis and overall survival of 50 glioma patients 39. Similar with the results derived from the TCGA dataset, old glioma patients were associated with low tumor overall survival in GSE43378 dataset (P=0.0012) (Figure 3A). Compared with the young glioma patients, genes up or down regulated in old glioma patients were also identified (fold change >2). Heatmap demonstrated 165 age related genes in glioma patients derived from GSE43378 dataset (Figure 3B). Among them, 32 genes were also differentially expressed in LGG patients derived from TCGA dataset (Figure 3C). We then focused on those 32 genes for our further studies.

First, the regulatory network of those 32 age related genes was constructed using Cytoscape (Figure 3D). EMP3 was one of the hub genes in this network. Previous results suggested that EMP3 was a critical biomarker in glioma prognosis 28-32. EMP was connected with TIMP1 and SERPINE1 and SERPINE1 was connected with IGFBP2 (Figure 3D). It had been reported that IGFBP2 could promote glioma development and was also a critical biomarker in glioma prognosis 33-35. However, the prognostic effects of TIMP1 and SERPINE1 in LGG patients were unclear. We showed that IGFBP2, EMP3, TIMP1 and SERPINE1 were all highly expressed in old LGG patients compared with young LGG patients in TCGA dataset (Figure 3E). Similarly, old glioma patients were with high expressions of those genes in GSE43378 dataset (Figure 3F).

### DNA methylation profiles of age related genes in LGG patients

**Figure 4:**
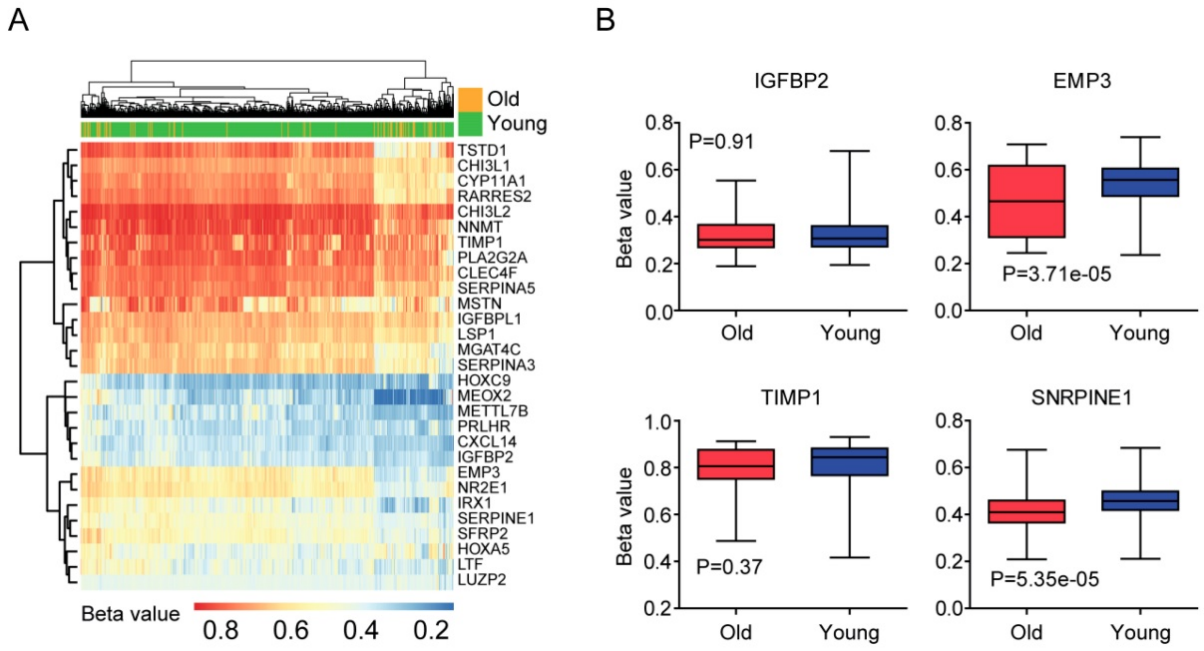
DNA methylation profiles of age related genes in LGG patients. (A) Unsupervised clustering heatmap demonstrated the DNA methylation intensity (Beta value) of the 32 age related genes in old and young LGG patients. (B) Box plots showed the IGFBP2, EMP3, TIMP1 and SERPINE1 DNA methylation intensity (Beta value) in TCGA LGG patients.

Secondly, we tested the DNA methylation of age related genes in old LGG patients. Heatmap demonstrated the DNA methylation intensity (Beta value) of the 32 age related genes (Figure 4A). We found that some genes, for example TIMP1, NNMT and CHI3L2 genes were hyper-methylated in LGG patients, while, HOXC9, MEOX2, METTL7B, PRLHR, CXCL14 and IGFBP2 genes were hypo-methylated in LGG patients (Figure 4A).

Previous results suggested that the expression of EMP3 in glioma patients was controlled by DNA methylation 29. We found that DNA methylation intensity of EMP3 gene was lower in old LGG patients, compared with young LGG patients (P=3.71e-05) (Figure 4B). Similarly, age related gene SERPINE1 was hypo-methylated in old LGG patients, compared with young LGG patients (P=5.35e-05) (Figure 4B). We speculated that the hypo-methylations of EMP3 and SERPINE1 were contributing to the high expressions of EMP3 and SERPINE1 in old LGG patients (Figure 3E and 3F). However, we did not find significant different methylation intensity of IGFBP2 and TIMP1 between old and young LGG patients (Figure 4B). And how IGFBP2 and TIMP1 genes were increased in old LGG patients still needed further studies.

### Expression profiles of age related genes in normal and glioma tissues

**Figure 5:**
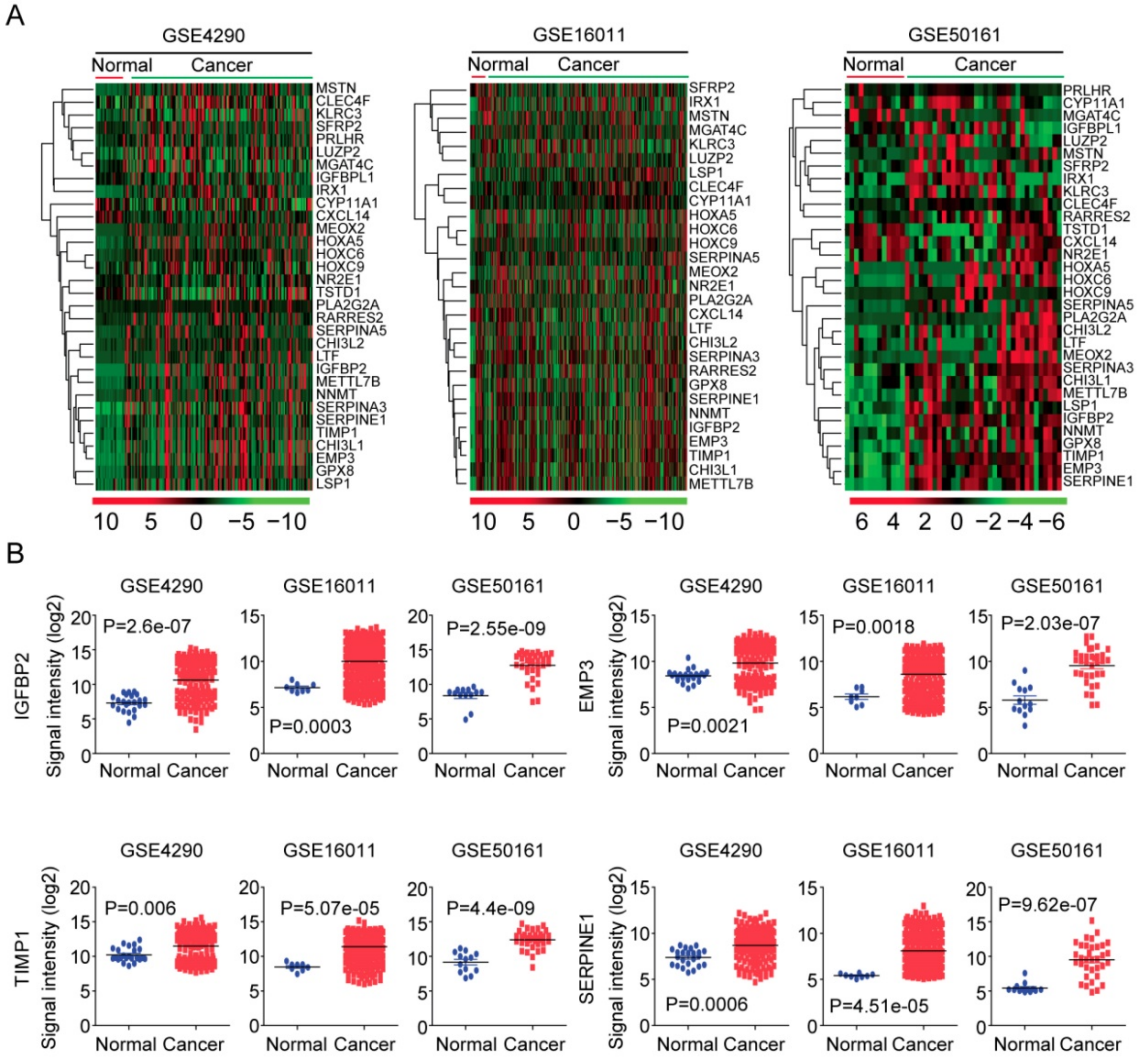
Expression profiles of age related genes in normal and glioma tissues. (A) Unsupervised clustering heatmaps demonstrated the expressions of the 32 age related genes in normal brain tissues and glioma tissues in GSE4920, GSE16011 and GSE50161 three datasets. (B) Box plots showed the IGFBP2, EMP3, TIMP1 and SERPINE1 expressions (log2 signaling intensity) in normal brain tissues and glioma tissues in GSE4920, GSE16011 and GSE50161 three datasets.

Next, using GSE4920 36, GSE16011 37 and GSE50161 38 datasets, we tested the expressions of the 32 age related genes in normal brain tissues and glioma tumor tissues. As illustrated in the heatmaps, most age related genes were up regulated in glioma tumor tissues (Figure 5A). Interestingly, IGFBP2, EMP3, TIMP1 and SERPINE1 were clustered into a small group of genes in GSE4920, GSE16011 and GSE50161 three datasets. And as illustrated in the box plots, IGFBP2, EMP3, TIMP1 and SERPINE1 were all highly expressed in glioma tumor tissues, compared with the normal brain tissues (Figure 5B).

### Prognostic significance of age related genes in glioma patients

Next, using univariate cox regression analysis, we determined the prognostic significance of age related genes in glioma patients in both TCGA and GSE43378 datasets. 25 genes out of 32 genes were significantly associated with tumor overall survival in both TCGA and GSE43378 datasets (P< 0.05) (Table 2), suggesting that the age related genes played important roles in determining the overall survival of LGG patients. For example, high IGFBP2, EMP3, TIMP1 and SERPINE1 expressions were all positively associated with the overall survival of LGG patients, while, high SFRP2, IRX1, KLRC3 and LUZP2 expressions were all negatively associated with the overall survival of LGG patients (Table 2).

**Figure 6:**
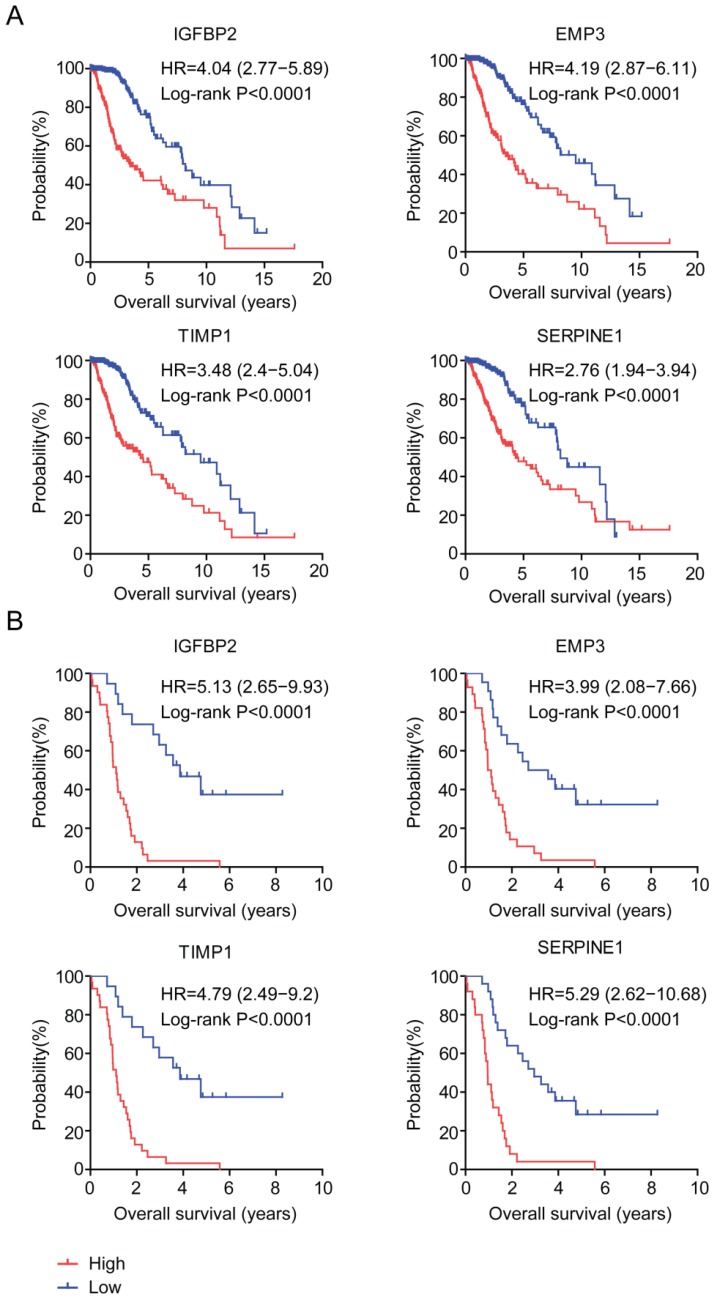
Prognostic significance of age related genes in glioma patients. (A) Relationships of IGFBP2, EMP3, TIMP1 and SERPINE1 expressions and overall survival were analyzed in TCGA dataset. Kaplan-Meier survival analysis was used to compare IGFBP2, EMP3, TIMP1 and SERPINE1 highly expressed LGG patients (red) with IGFBP2, EMP3, TIMP1 and SERPINE1 lowly expressed LGG patients (blue). P values were generated from Log-rank test. (B) Relationships of IGFBP2, EMP3, TIMP1 and SERPINE1 expressions and overall survival were analyzed in GSE43378 dataset.

The prognostic significance of age related genes IGFBP2, EMP3, TIMP1 and SERPINE1 were further demonstrated in Kaplan-Meier survival analysis in glioma patients. Our results showed that, high expressions of IGFBP2, EMP3, TIMP1 and SERPINE1 genes were unfavorable prognostic markers in TCGA (Figure 6A) and GSE43378 patients (Figure 6B). LGG patients with higher expression of IGFBP2, EMP3, TIMP1 and SERPINE1 had worse prognosis than patients with low expression of those genes.

### Combined EMP3 and SERPINE1 genes in glioma overall survival prediction

**Figure 7:**
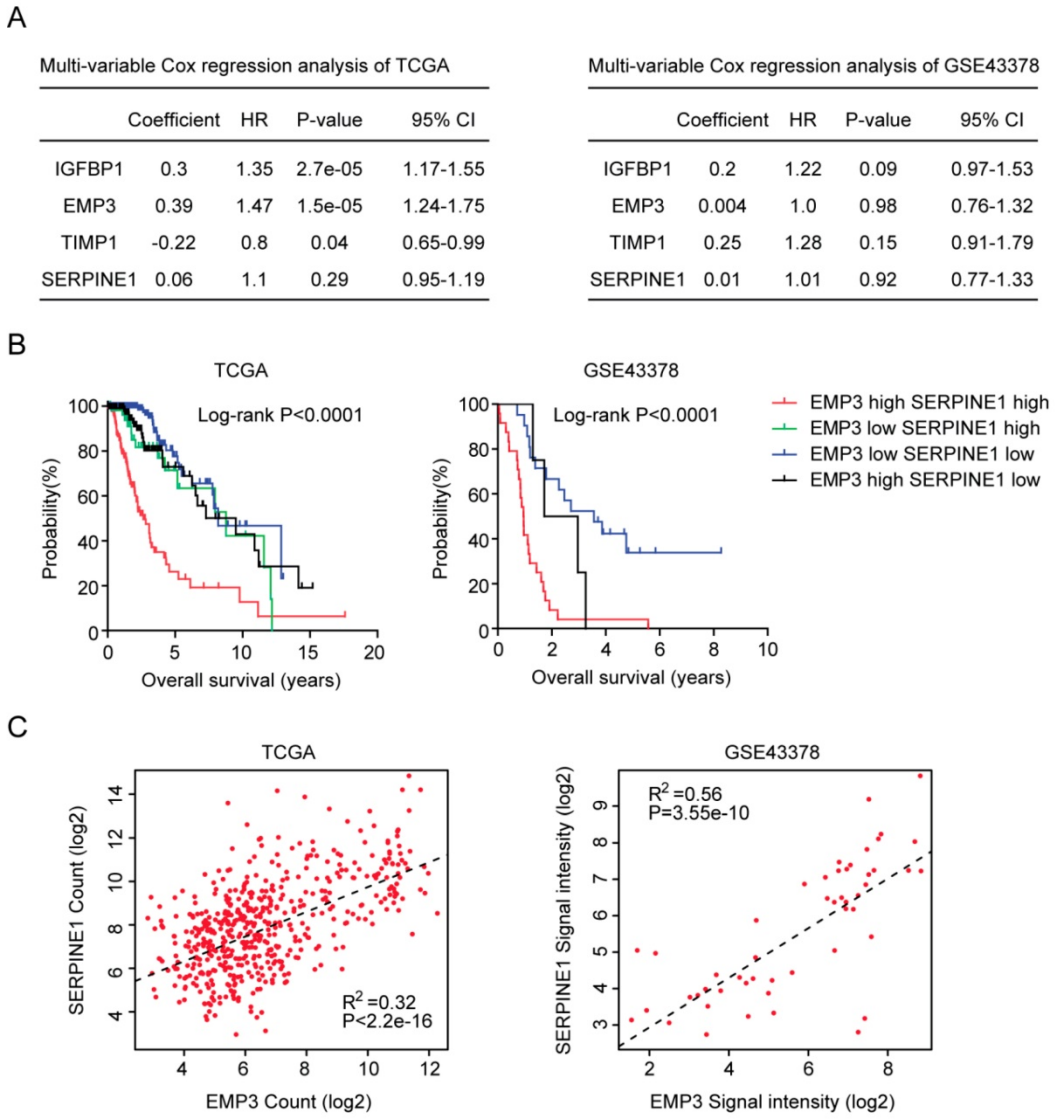
Combined EMP3 and SERPINE1 genes in glioma overall survival prediction. (A) Multivariate cox regression was used to test the relationships of IGFBP2, EMP3, TIMP1 and SERPINE1 expressions and overall survival in glioma patients in TCGA and GSE43378 datasets. (B) Kaplan-Meier plotters demonstrated the different overall survival of glioma patients with high expressions of EMP3 and SERPINE1 and glioma patients with low expressions of EMP3 or SERPINE1 in TCGA and GSE43378 datasets. Log-rank test was used to determine the P values. (C) Spearman correlation between EMP3 and SERPINE1 expressions in TCGA and GSE43378 datasets was determined.

We also used multivariate cox regression to reveal the connection of IGFBP2, EMP3, TIMP1 and SERPINE1 genes in determining the clinical overall survival of LGG patients. We found that IGFBP2, EMP3 and TIMP1 were independent prognostic markers in TCGA LGG patients (Figure 7A). However, in GSE43378 dataset, IGFBP2, EMP3, TIMP1 and SERPINE1 genes were interconnected with each other, and those genes were not independent prognostic markers (Figure 7A).

So, in both TCGA and GSE43378 datasets, SERPINE1 was not an independent prognostic marker. We thought that the combination of SERPINE1 with other age related genes, particular EMP3, could be used as better prognostic marker in LGG patients. To test this hypothesis, the average expression level of EMP3 and SERPINE1 was calculated. Based on the average expression level of EMP3 and SERPINE1, glioma patients were divided into four groups. We found that patients with both high EMP3 and SERPINE1 expressions were particular with lowest overall survival in glioma patients (Figure 7B). Patients with the low expression of EMP3 or SERPINE1 had better clinical outcomes (Figure 7B). At last, we tried to determine the connection between EMP3 and SERPINE1. Spearman correlation demonstrated a high correlation between EMP3 and SERPINE1 in glioma expression TCGA and GSE43378 datasets ((Figure 7C).

### Validation of age related genes in Chinese LGG patients

**Figure 8:**
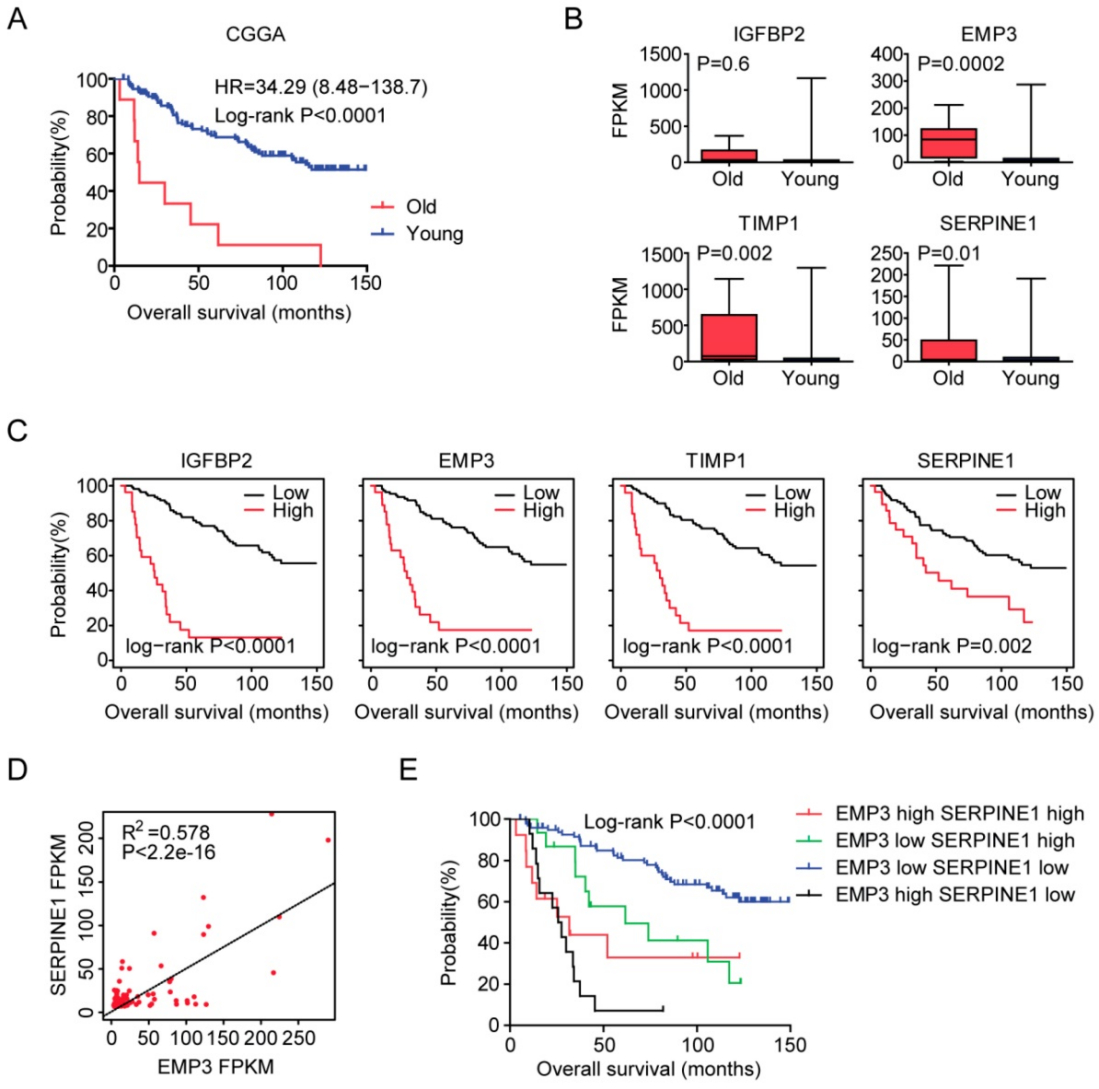
Validation of age related genes in Chinese LGG patients derived from CGGA dataset. (A) Overall survival was analyzed in old (red) and young (blue) LGG patients in CGGA dataset. P value was generated from Log-rank test. (B) Box plots showed the IGFBP2, EMP3, TIMP1 and SERPINE1 expressions (FPKM) in CGGA LGG patients. (C) Relationships of IGFBP2, EMP3, TIMP1 and SERPINE1 expressions and overall survival were analyzed in CGGA dataset. Kaplan-Meier survival analysis was used to compare IGFBP2, EMP3, TIMP1 and SERPINE1 highly expressed LGG patients (red) with IGFBP2, EMP3, TIMP1 and SERPINE1 lowly expressed LGG patients (black). P values were generated from Log-rank test. (D) Spearman correlation between EMP3 and SERPINE1 expression in CGGA dataset was determined. (E) Kaplan-Meier plotters demonstrated the different overall survival of glioma patients with high expressions of EMP3 and SERPINE1 and glioma patients with low expressions of EMP3 or SERPINE1 in CGGA dataset. Log-rank test was used to determine the P values.

At last, we tried to validate the age related genes in Chinese LGG patients. 140 LGG patients were collected from the Chinese Glioma Genome Atlas (CGGA) dataset. Similar with the previous results, old LGG patients (older than 60 years) were associated with low tumor overall survival in CGGA dataset (P<0.0001) (Figure 8A). Also, EMP3, TIMP1 and SERPINE1 were all highly expressed in old LGG patients compared with young LGG patients (Figure 8B). However, we did not find significant different expression level of IGFBP2 between old and young LGG patients. We also showed that the high expressions of IGFBP2, EMP3, TIMP1 and SERPINE1 genes were associated with the unfavorable prognosis of LGG patients in CGGA dataset (Figure 8C). Moreover, EMP3 and SERPINE1 were highly correlated with each other (Figure 8D) and the combination of EMP3 and SERPINE1 genes had better survival prediction in Chinese patients with LGG (Figure 8E). All those results were quietly similar with the results derived from TCGA and GSE43378 datasets.

## Discussion

With the increasing of age, high incidence of cancer is occurred. For example, more than 70% of LUSC, BLCA and COAD patients are diagnosed in old adults (Figure 1A). However, some tumors are particularly developed in young adults, like LGG and CESC. Nearly 90% LGG and CESC patients are developed under 60 years old (Figure 1A). Those observations highlight the age related disparities in different tumor types. Although, in general, old tumor patients have worse prognosis than young tumor patients (Figure 1B), age only is not always associated with the overall survival in all tumor types 40, 41. For example, there is no difference in the clinical overall survival in old LUAD, LIHC, ESCA and COAD patients compared with young LUAD, LIHC, ESCA and COAD patients (Figure 1C). However, LGG is one of the most age related tumor type. Age is most associated with the overall survival of LGG patients than other types of tumor patients (Figure 1C). Moreover, there are most age related genes in old LGG patients (Figure 2). Those observations intrigue great interests for the further studies of age related molecular profiles in LGG patients.

First, the age related genes in LGG patients are identified from TCGA dataset (Figure 2), and validated using GSE43378 and CGGA datasets (Figure 3 and 8). DNA methylation intensity (Figure 4A and 4B) and prognostic effects (Table 2) of those age related genes are also demonstrated. Furthermore, the expression profiles of age related genes in normal and glioma tissues are revealed (Figure 5A). Base on those results, we find four age related genes IGFBP2, EMP3, TIMP1 and SERPINE1 may represent suitable biomarkers for prognostic strategies of LGG patients.

The prognostic effects of EMP3 28-32 and IGFBP2 33-35 in glioma patients are extensively studied. Here, we find two EMP3 and IGFBP2 associated genes TIMP1 and SERPINE1 are also suitable biomarkers for prognosis of glioma patients. EMP3, IGFBP2, TIMP1 and SERPINE1 are highly expressed in old LGG patients (Figure 3E and 3F). In LGG tumor tissues, EMP3, IGFBP2, TIMP1 and SERPINE1 are all highly expressed, compared with normal brain tissues (Figure 5B). Furthermore, high expressions of IGFBP2, EMP3, TIMP1 and SERPINE1 are associated with low overall survival of LGG patients (Figure 6). Interestingly, EMP3 and SERPINE1 are connected with each other and the combination of EMP3 and SERPINE1 genes have better prognostic effects in glioma patients (Figure 7B and 7C).

Our results also show some inner mechanisms of how EMP3 and SERPINE1 are increased in old LGG patients. We find that EMP3 and SERPINE1 are hypo-methylated in old LGG patients, compared with young LGG patients (Figure 4B). Although the expression of IGFBP2 is controlled by epigenetic DNA methylation in glioma patients 42, we find no significant different methylation intensity of IGFBP2 and TIMP1 genes between old and young LGG patients (Figure 4B). So, how IGFBP2 and TIMP1 genes are increased in old LGG patients still need further studies.

Overall, our results provide deep understandings of how age and age related genes influence the clinical overall survival of LGG patients. Although clinical further validations are needed, our analysis suggest that IGFBP2, EMP3, TIMP1 and SERPINE1 genes, particular the combination of EMP3 and SERPINE1, could be used as biomarkers to predict the overall survival of LGG patients.

## Conclusions

Age related genes IGFBP2, EMP3, TIMP1 and SERPINE1 have significant prognostic effects in LGG patients.

## Supplementary Material

Supplementary table.

## Figures and Tables

**Table 1:** Summary of tumor types and the number of tumor patients in TCGA dataset used in this study.

Abbreviations	Tumor type	Overall	Old	Young
BLCA	Bladder Urothelial Carcinoma	408	301	107
BRCA	Breast Invasive Carcinoma	1082	482	600
CESC	Cervical Squamous Cell Carcinoma	294	54	240
COAD	Colon Adenocarcinoma	441	306	135
ESCA	Esophageal Adenocarcinoma	185	93	92
GBM	Glioblastoma Multiforme	596	272	324
HNSC	Head and Neck Squamous Cell Carcinoma	525	267	258
KIRC	Kidney Renal Clear Cell Carcinoma	535	269	266
KIRP	Kidney Renal Papillary Cell Carcinoma	286	152	134
LGG	Brain Lower Grade Glioma	511	61	450
LIHC	Liver Hepatocellular Carcinoma	371	195	176
LUAD	Lung Adenocarcinoma	498	339	159
LUSC	Lung Squamous Cell Carcinoma	492	385	107
OV	Ovarian Serous Cystadenocarcinoma	580	258	322
PAAD	Pancreatic Adenocarcinoma	184	123	61
PRAD	Prostate Adenocarcinoma	497	274	223
SARC	Sarcoma	261	133	128
SKCM	Skin Cutaneous Melanoma	107	66	41
STAD	Stomach Adenocarcinoma	413	276	137
UCEC	Uterine Corpus Endometrial Carcinoma	544	335	209
PAN-Cancer		8810	4641	4169

**Table 2:** Univariate cox regression was used to reveal the prognostic significance of age related genes in glioma patients.

	TCGA				GSE43378			
symbol	Coefficient	HR	P-value	95% CI	Coefficient	HR	P-value	95% CI
IGFBP2	0.44	1.55	<2e-16	1.43-1.68	0.37	1.44	3.38e-06	1.24-1.68
HOXA5	0.34	1.35	<2e-16	1.27-1.44	0.25	1.28	0.0013	1.1-1.49
SERPINA5	0.25	1.28	<2e-16	1.21-1.36	0.365	1.43	4.50e-05	1.2-1.7
RARRES2	0.23	1.26	1.2E-09	1.17-1.36	0.24	1.27	0.0012	1-1.46
TIMP1	0.37	1.44	<2e-16	1.33-1.57	0.42	1.52	1.07e-06	1.28-1.79
PLA2G2A	0.2	1.22	5.3e-11	1.15-1.29	0.31	1.37	4.30e-05	1.18-1.59
CYP11A1	0.16	1.17	0.00455	1.05-1.30	-0.03	0.97	0.79	0.81-1.18
MEOX2	0.29	1.3	<2e-16	1.25-1.41	0.26	1.29	0.0031	1.1-1.53
HOXC9	0.39	1.46	<2e-16	1.35-1.58	0.18	1.19	0.01	1.04-1.37
HOXC6	0.31	1.36	<2e-16	1.27-1.46	0.17	1.18	0.0097	1.04-1.34
CXCL14	0.1	1.11	0.0177	1.02-1.2	0.21	1.24	0.022	1.03-1.48
NR2E1	0.35	1.42	8.8e-01	1.27-1.6	0.15	1.17	0.13	0.96-1.42
LSP1	0.45	1.57	2.1e-13	1.39-1.77	0.47	1.59	0.0031	1.17-2.16
SERPINA3	0.29	1.33	2.7e-13	1.23-1.44	0.25	1.28	0.00031	1.12-1.46
NNMT	0.3	1.34	7.2e-16	1.25-1.44	0.25	1.29	1.04e-05	1.15-1.44
LTF	0.16	1.16	1.8e-10	1.11-1.22	0.14	1.15	0.000185	1.07-1.24
GPX8	0.43	1.53	<2e-16	1.39-1.68	0.41	1.5	2.90e-06	1.27-1.78
EMP3	0.45	1.56	<2e-16	1.44-1.7	0.37	1.44	4.19e-05	1.21-1.72
SERPINE1	0.29	1.33	1.9e-11	1.23-1.45	0.37	1.45	7.70e-06	1.23-1.7
METTL7B	0.49	1.5	<2e-16	1.38-1.64	0.44	1.55	4.39e-07	1.3-1.84
CHI3L2	0.26	1.29	1.9e-10	1.19-1.39	0.27	1.3	0.00064	1.12-1.52
CHI3L1	0.27	1.31	<2e-16	1.24-1.38	0.27	1.31	2.81e-05	1.15-1.48
TSTD1	0.35	1.42	1.7E-12	1.29-1.57	0.15	1.16	0.12	0.96-1.4
IGFBPL1	-0.13	0.88	0.0039	0.81-0.96	-0.19	0.82.	0.082	0.66-1.02
MGAT4C	-0.31	0.73	1.2e-11	0.67-0.8	-0.35	0.71	0.0001	0.59-0.84
MSTN	-0.03	0.97	0.397	0.9-1.04	-0.26	0.77	0.0033	0.65-0.92
SFRP2	-0.25	0.78	<2e-16	0.73-0.82	-0.13	0.88	0.031	0.78-0.99
CLEC4F	-0.31	0.74	2.8e-08	0.66-0.82	-0.13	0.88	0.19	0.72-1.1
PRLHR	-0.23	0.79	6.2e-14	0.74-0.84	-0.24	0.78	0.0011	0.68-0.91
IRX1	-0.04	0.96	0.26	0.9-1.03	-0.02	0.98	0.82	0.84-1.15
KLRC3	-0.26	0.77	1.8e-13	0.72-0.83	-0.18	0.83	0.017	0.71-0.97
LUZP2	-0.32	0.73	<2e-16	0.67-0.78	-0.35	0.71	0.0003	0.58-0.85

## Abbreviations

TCGA: The Cancer Genome Atlas
GEO: Gene Expression Omnibus
CGGA: Chinese Glioma Genome Atlas
BLCA: Bladder Urothelial Carcinoma
BRCA: Breast Invasive Carcinoma
CESC: Cervical Squamous Cell Carcinoma
COAD: Colon Adenocarcinoma
ESCA: Esophageal Adenocarcinoma
GBM: Glioblastoma Multiforme
HNSC: Head and Neck Squamous Cell Carcinoma
KIRC: Kidney Renal Clear Cell Carcinoma
KIRP: Kidney Renal Papillary Cell Carcinoma
LGG: Brain Lower Grade Glioma
LIHC: Liver Hepatocellular Carcinoma
LUAD: Lung Adenocarcinoma
LUSC: Lung Squamous Cell Carcinoma
OV: Ovarian Serous Cystadenocarcinoma
PAAD: Pancreatic Adenocarcinoma
PRAD: Prostate Adenocarcinoma
SARC: Sarcoma
SKCM: Skin Cutaneous Melanoma
STAD: Stomach Adenocarcinoma
UCEC: Uterine Corpus Endometrial Carcinoma
